# Genetic diversity and differentiation of *Daphnia galeata* in the middle and lower reaches of the Yangtze River, China

**DOI:** 10.1002/ece3.5737

**Published:** 2019-10-22

**Authors:** Qi Liu, Daogui Deng, Kun Zhang, Ping He, Yuchen Sun, Tingting Zhang, Wei Yang, Wei Liu

**Affiliations:** ^1^ School of Life Science Huaibei Normal University Huaibei China

**Keywords:** *Daphnia galeata*, genetic differentiation, genetic diversity, haplotype, the middle and lower reaches of the Yangtze River

## Abstract

Mitochondrial *16S* rDNA and *CO* I gene were used as molecular markers for the analysis of the genetic diversity and differentiation of *Daphnia galeata* populations in nine water bodies in the middle and lower reaches of the Yangtze River. In the combined *16S* rDNA and *CO* I gene sequences, 54 variable sites and 44 haplotypes were observed among 219 individuals belonging to nine *D. galeata* populations. Average haplotype diversity and nucleotide diversity were, respectively, 0.72% and 0.56%. The *F*‐statistics (*F*_ST_) value of the *D. galeata* populations was 0.149. According to the results of the neutral test, *D. galeata* in the middle and lower reaches of the Yangtze River had experienced a bottleneck effect in the history. Molecular variance analysis indicated that the genetic differentiation of the *D. galeata* populations mainly occurred within populations (85.09%). Greater genetic differentiations of *D. galeata* among individuals within populations appeared in the populations from the Huaihe River basin, whereas smaller genetic differentiations occurred in the populations from the middle reaches of the Yangtze River. Strong gene flows were all observed between Group I (four populations from the middle reaches of the Yangtze River) and Group ΙΙ (three populations from the middle and lower reaches of the Yangtze River), and Group ΙΙΙ (two populations from the Huaihe River basin). The effective migration rates (*M*) were 851.49 from Group I to Group ΙΙ and 685.96 from Group I to Group ΙΙΙ, respectively. However, no significant relationship was observed between the genetic differentiation and geographical distance of the nine populations (*r* = .137, *p* > .05). Results suggested that the genetic differentiation of *D. galeata* in the water bodies in the middle and lower reaches of the Yangtze River resulted mainly from geographical isolation.

## Graphical abstract

In this study, greater genetic differentiations of *Daphnia galeata* among individuals within populations appeared in the populations from the Huaihe River basin whereas smaller genetic differentiations occurred in the populations from the middle reaches of the Yangtze River. Strong gene flows were all observed between Group I (four populations from the middle reaches of the Yangtze River) and Group II (three populations from the middle and lower reaches of the Yangtze River), and Group III (two populations from the Huaihe River basin). The effective migration rates (*M*) were 851.49 from Group I to Group II and 685.96 from Group I to Group III, respectively. However, no significant relationship was observed between the genetic differentiation and geographical distance of the nine populations (*r* = .137, *p* > .05). Results suggested that the genetic differentiation of *D. galeata* in the water bodies in the middle and lower reaches of the Yangtze River resulted mainly from geographical isolation.

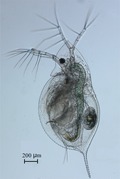

## 1 INTRODUCTION

Cladocerans (Crustacean: Cladocera) are important components of aquatic ecosystems (Jiang & Du, 1979). The genus of *Daphnia* is a key component of cladocerans, which are characterized by their wide distribution, easy cultivation, fast propagation (Adamowicz & Purvis, 2005; Benzie, 2005; Lampert & Kinne, 2011), and sensitivity to environmental changes (Su, 2013). *Daphnia* has become an efficient biological model for the biogeography of freshwater zooplankton in recent decades (Ma et al., 2015). *Daphnia galeata*, which belongs to the *Daphnia longispina* complex (Petrusek et al., 2008) and is a common species of *Daphnia*, is typically found in relatively warm and eutrophic lakes or reservoirs (Spaak, Fox, & Hairston, 2012) and mainly distributed in Asia, Europe, and North America (Ishida et al., 2011; Keller, Wolinska, Manca, & Spaak, 2008; Taylor & Hebert, 2010; Yin, Gießler, Griebel, & Wolinska, 2014). In China, *D. galeata* is widespread in lakes and reservoirs (Jiang & Du, 1979; Ma et al., 2015; Xie, Xu, Ren, Xu, & Han, 2015; Xu et al., 2018). Wei et al. (2015) examined the genetic structure of eight populations of the *D. longispina* complex in Eastern China and found that only *D. galeata* appeared in the investigated lakes. In addition, *D. galeata* was also detected as the main species in eutrophic lakes located in central and eastern China (Ma et al., 2015; Xu et al., 2018). Usually, *Daphnia* species have high dispersal capabilities (Louette & De Meester, 2005). How the adaptability of *Daphnia* to newly ecological environments or how to evolve in a watershed system is not well known.

The genetic diversity and phylogenetic evolution of *Daphnia* species have been extensively studied on the basis of molecular markers. Based on the haplotype diversity (*Hd*) and nucleotide diversity (*π*) of mitochondrial gene sequences, Grant and Bowen (1998) classified the genetic diversity of marine organisms into four types: (a) low *Hd* (<0.5) and low *π* (<0.5%), suggesting recent population bottleneck or founder event; (b) high *Hd* (>0.5) and low *π* (<0.5%), indicating an expansion after a period of low effective population size and enhanced retention of new mutations; (c) low *Hd* (<0.5) and high *π* (>0.5%), suggesting the secondary contact between isolated populations or a strong bottleneck in a formerly large and stable population; (d) high *Hd* (>0.5) and high *π* (>0.5%), denoting the high level of divergence between haplotypes. Hebert, Witt, and Adamowicz (2003) observed that long‐time geographical segregation resulted in genetic differences among *Daphnia ambigua* populations in five different geographical regions. Xu et al. (2018) found that the genetic distance differences between 10 *Daphnia* distributed in different water bodies of the Tibetan Plateau were 9.25%–30.71%. A high genetic differentiation was observed among *Daphnia magna* species in Europe, North America, and Japan (De Gelas & De Meester, 2005), suggesting an intercontinental diffusion of the species. Compared with the *D. magna* populations living in the medium‐sized and stable ponds of southern or central Europe, those located in small rock pools of northern Europe have significantly low genetic diversity and high genetic differentiation (Walser & Haag, 2012). By examining 49 screened populations of *D. longispina* and 77 populations of *D. magna*, Haag, Riek, Hottinger, Pajunen, and Ebert (2005) found that local genetic diversity increased with population age, whereas pairwise differentiation among populations decreased. Straughan & Lehman (2000) found that the genetic differences of *Daphnia pulex* complex among different lakes were affected by river basin location, regional physical geography, bird migratory flyways, and lake trophic status. Meanwhile, *D. longispina* complex, *D. galeata*, and *Daphnia dentifera* living in the New North District had introgression in Quaternary glacial activity (Ishida et al., 2011). *Daphnia galeata* was mainly distributed in eutrophic lakes in low‐altitude areas in central and eastern China, whereas *D. dentifera* was distributed in high‐altitude oligotrophic lakes in the Tibetan Plateau in western China (Ma et al., 2015).

The Yangtze River Basin is a concentrated area of freshwater lakes in China, where many lakes have become eutrophic (Deng et al., 2008; Huang, Gao, & Zhang, 2016; Qin, 2002; Wu, Qin, & Yu, 2016). Two major river systems, the Yangtze River and the Huaihe River, run through this basin. Historically, the Huaihe River drained directly into the Yellow Sea, but it is now connected to the lower reaches of the Yangtze River after many floods (Wu, Wang, et al., 2019a). Along the Yangtze River Basin, many tributaries of the river and lakes are distributed, which is an ideal habitat for studying the genetic differentiation of *Daphnia*. Wang, Zhang, et al. (2016) observed the genetic differentiation among *D. pulex* populations distributed in 10 water regions in the middle and lower reaches of the Yangtze River. Wang (2016) also found genetic differences among *Daphnia similoides sinensis* from the hatching of resting eggs in different sedimentary layers of Lake Chaohu. Wu, Wang, et al. (2019a) found that *D. similoides sinensis* from seven water bodies located in the middle and lower reaches of the Yangtze River had evolved into two clades, namely the Yangtze River clade and the Huaihe River clade. In Lake Junshan, Wu, Zhang, et al. (2019b) observed that there were obviously genetic differences of *D. similoides sinensis* before and after the construction of artificial dam. Similarly, some genetic differentiation of *D. galeata* populations from lakes or reservoirs located in the upper or lower reaches of the Yangtze River was found, and these differentiations were not related to geographical distance (Wei et al., 2015; Xie et al., 2015). Although there were some investigations on the genetic differentiation of *Daphnia* in the Yangtze River, it was fragmented and unsystematic for *D. galeata* in the area.

In this study, we selected *16S* rDNA and *CO* I gene as molecular markers to investigate the genetic diversity and differentiation of *D. galeata* in nine water bodies in the middle and lower reaches of the Yangtze River and explore the relationship between genetic differentiation and geographical distribution pattern. This study will increase understanding of the genetic structure and global phylogenetic evolution of *D. galeata*.

## 2 MATERIALS AND METHODS

### 2.1 Animal culture

**Figure 1:**
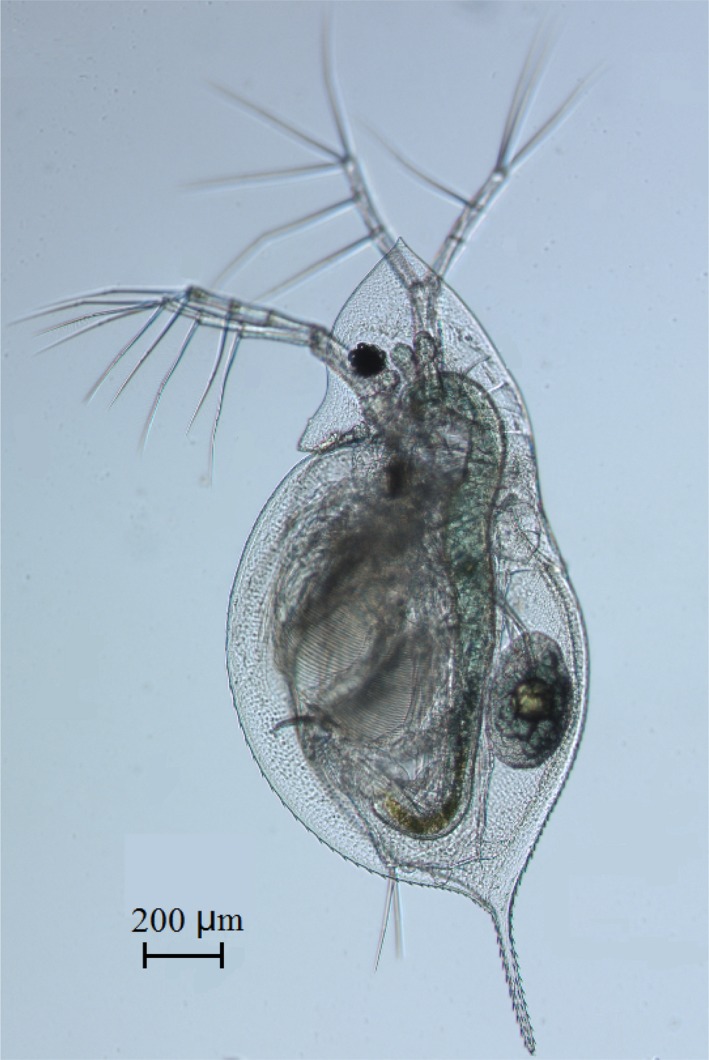
The photograph of *Daphnia galeata* female

**Figure 2:**
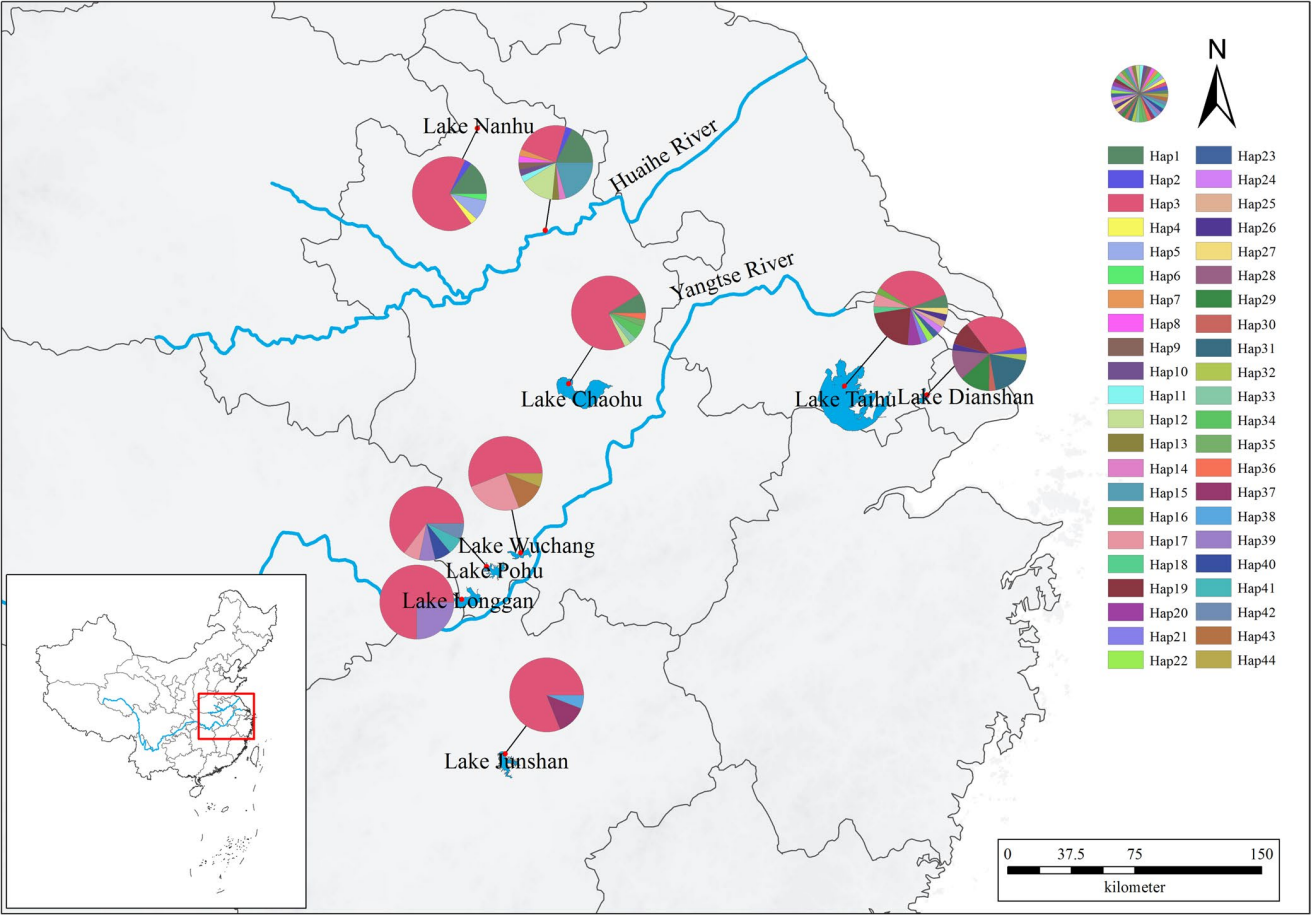
Relative frequencies and geographical distribution of haplotypes in the *Daphnia galeata* populations from nine water bodies located in the middle and lower reaches of the Yangtze River based on the combined sequences of *16S* rDNA and *CO* I genes

*Daphnia galeata* (Figure 1) was collected from two water bodies of Huaihe River basin (Lake Nanhu, Huaihe River section in Bengbu city) and seven lakes in the middle and lower reaches of the Yangtze River (Lake Junshan, Lake Longgan, Lake Pohu, Lake Wuchang, Lake Chaohu, Lake Taihu, and Lake Dianshan) (Table 1, Figure 2). The identification of *D. galeata* was according to the methods of Jiang and Du (1979) and Benzie (2005). The collected *D. galeata* individuals were monoclonally cultured in an intelligent lighting incubator (Ningbo Saifu), with the illumination of 12 hr light: 12 hr dark at (25 ± 1)°C, and 4–6 mature females per individual were selected for DNA extraction. The culture medium was changed every day, and *D. galeata* were fed with *Scenedesmus obliquus* of 2 × 10^5^ cells/ml. The culture medium was aerated tap water over 48 hr, filtered by boiling and cooling for reserve.

**Table 1:** Geographical information and code of the *Daphnia galeata* populations from nine water bodies in the middle and lower reaches of the Yangtze River

Sampling sites	Code	Latitude (N)	Longitude (E)	Surface area (km^2^)	Trophic status	No. individuals
Lake Nanhu Anhui province	HN	33°89′–33°90′	116°79′–116°80′	2.1	Mesotrophic Deng et al., 2010	33
Huaihe River section in Bengbu city Anhui province	AH	32°56′	117°20′	–	Eutrophic Zuo, Chen, Zhang, Dou, & Liu, 2017	34
Lake Taihu Jiangsu province	TH	30°55′–31°51′	119°52′–120°36′	2,338	Eutrophic He et al., 2018	36
Lake Dianshan, Shanghai city	DS	31°07′–31°19′	120°92′–121°02′	62	Eutrophic Liu, Wang, et al., 2018	31
Lake Chaohu Anhui province	CH	31°25′–31°43′	117°17′–117°52′	753	Eutrophic Zhang, 2016	35
Lake Junshan Jiangxi province	JS	28°25′–28°37′	116°17′–116°23′	213	Mesotrophic Liu, He, et al., 2018	16
Lake Longgan Anhui province	LG	29°52′–29°58′	116°01′–116°16′	223	Mesotrophic Meng et al., 2013	4
Lake Pohu Anhui province	PH	30°06′–30°11′	116°20′–116°32′	233	Mesotrophic Du, Zhang, Fan, Zhi, & Xiao, 2015	14
Lake Wuchang Anhui province	WC	30°14′–30.20′	116°36′–116°53′	105	–	16

### 2.2 DNA extraction and PCR amplification

The genomic DNA of *D. galeata* was extracted by using TIANamp Micro DNA Kit (Tiangen). Each *D. galeata* body was crushed with a sterile 10 μl tip before extraction because the chitin carapace of *D. galeata* hindered the digestion of internal organs by proteinase K. The concentration of DNA extraction was measured by a spectrophotometer (Biofuture) and then stored at −20°C for subsequent analyses.

The mitochondrial *16S* rDNA was amplified with the L2510 5′ to 3′ (CGCCTGTTTAACAAAAACAT) and H3059 5′ to 3′ (CCGGTCTGAACTCAGATCATGT) (Bouchon, Souty‐Grosser, & Raimond, 1994). The mitochondrial *CO* I gene was amplified with the LCO1490 5′ to 3′ (GGTCAACAAATCATAAAGATATTGG) and HCO2198 5′ to 3′ (TAAACTTCAGGGTGAC CAAAAAATCA) (Xu et al., 2014).

PCR reaction (25 μl) contained 1.0 μl of genomic DNA (approximately 100 ng/μl), 2.5 μl of 10× LA‐Taq Buffer II, 4.0 μl of dNTPs (2.5 mM) (Shanghai Shenggong), 0.5 μl of Mg^2+^ (25 mM), 1.0 μl of each upper and lower primers (10 mM) (Shanghai Shenggong), 0.25 μl of DNA polymerase TaKaRa‐LA‐Taq (5 U/μl) (Clontech), and 14.75 μl of double‐distilled H_2_O. The conditions of the *16S* rDNA amplification included an initial denaturing step of 3 min at 95°C, 35 cycles of 45 s at 95°C, 45 s at 50°C, 55 s at 72°C, and a final extension of 72°C for 10 min. The conditions of the *CO* I gene amplification included an initial denaturing step of 1 min at 95°C, 35 cycles of 40 s at 95°C, 40 s at 45°C, 1 min at 72°C, and a final extension of 72°C for 10 min. The amplified products were detected by 1% agarose gel electrophoresis, and the PCR products with clear target and brightness were sequenced by GenScript.

### 2.3 Data analyses

We compared the alignments of the *D. galeata 16S* rDNA and *CO* I gene sequences in the NCBI database to ensure the reliability of our sequences. Before the combined analysis of *16S* rDNA and *CO* I gene sequences, the homogeneity of the two gene sequences was tested by using PAUP*v4.0, and the results showed that the topologies of the two data sets were consistent (*p* = .14). Therefore, the two gene sequences can be combined.

The homologous alignment of *16S* rDNA and *CO* I gene sequences was performed by using Mega 7.0, and the missing sequences of the primer binding sites at both ends were removed after manual proofreading. The conversion/transversion ratio and base composition of the sequences were calculated with Mega 7.0. The genetic distances among the individuals and among the populations of *D. galeata* from the middle and lower reaches of the Yangtze River were calculated. A neighbor‐joining phylogenetic tree was constructed by using Mega 7.0 on the basis of the pairwise genetic distances among populations, and population groups were identified according to the genetic relationships among populations. *D. magna* (the Genbank serial numbers of *16S* rDNA and *CO* I gene sequences are KF993366.1 and KF993373.1, respectively) was used as outgroups. Principal component analysis (PCA) was performed on the genetic distance matrix among *D. galeata* individuals using Canoco 5.0. Molecular variation analysis was performed with Arlequin v3.5 (Excoffier & Lischer, 2010), and population genetic differentiation coefficients (*F*‐statistics, *F*_ST_) were also calculated. The “standard AMOVA analysis” method and 20,000 permutations were used to test the significance of the covariance components associated with different levels of genetic structure. A Mantel test was performed to identify significant correlations between population genetic differentiation coefficients (*F*_ST_) and geographical distances (km) using 1,000 randomization replicates in IBD.

DNASP 5.10 was employed to analyze the number of haplotype (*H*), haplotype diversity (*Hd*), nucleotide diversity (*π*), average number of nucleotide differences between whole sequences (*K*), Tajima's* D* value, and *Fu's Fs* value. Haplotype networks were constructed in Haploviewer (Salzburger, Ewing, & Haeseler, 2011). The directional gene flow was analyzed by Migrate‐n version 3.6 software using the strategy of Bayesian inference and constant mutation rate (Beerli, 2006; Beerli & Palczewski, 2010). The mutation‐scaled population sizes Ɵ and immigration rates *M* were calculated. The calculation formula of gene flow (Nm) is *X* × Nm = Ɵ × *M*, where *X* is the inheritance parameter and depends on the molecular marker, which is usually 1 for mt DNA data (Beerli, 2006; Beerli & Palczewski, 2010). A circle map of directional gene flow was drawn through the online version of the software CIRCOS (Krzywinski et al., 2009).

## 3 RESULTS

### 3.1 Characteristics of mitochondrial *16S* rDNA and *CO* I gene sequences in *D. galeata*

A total of 219 *D. galeata 16S* rDNA and *CO* I gene sequences were obtained. The sequences were aligned, and the unreliable bases at both ends were removed. The homologous sequences were 516 and 420 bp. The *16S* rDNA gene sequences had 516 identifiable sites, including 507 invariable sites, 9 variable sites, 1 single site, and 8 parsimony‐informative sites. The *CO* I gene sequences had 419 identifiable sites, including 374 invariable sites, 45 variable sites, 21 single sites, and 24 parsimony‐informative sites. The overall transition/transversion ratios of the *16S* rDNA and *CO* I gene sequences were 9.4 and 2.9, respectively. In the *16S* rDNA gene sequences, the average A, T/U, G, and C contents were 30.5%, 34.5%, 21.8%, and 13.2%, respectively. In the *CO* I gene sequences, the average A, T/U, G, and C contents were 23.4%, 33.6%, 21.9%, and 21.1%, respectively. The A + T contents (65% and 57%) were considerably higher than the C + G contents (35% and 43%) in the *16S* rDNA and *CO* I gene sequences.

Under the combined sequences of the *16S* rDNA and *CO* I genes, 936 base pairs and 935 identifiable sites, including 881 invariable sites, 54 variable sites, 22 single sites, and 32 parsimony‐informative sites, were found. The overall transition/transversion ratio was 4.4. The average A, T/U, G, and C contents were 27.3%, 34.1%, 21.8%, and 16.8%, respectively. The A + T content (61.4%) was significantly higher than the C + G content (38.6%).

### 3.2 Genetic diversity of *D. galeata* populations from the middle and lower reaches of the Yangtze River

A total of seven haplotypes (Hap1–Hap7) and 35 haplotypes (Hap1–Hap35) were, respectively, obtained (Figure 2a,b) in the *16S* rDNA and *CO* I gene sequences. Both indicated high levels of haplotype diversity (*Hd* = 0.37 and 0.67) (Table 2).

**Table 2:** Genetic diversity of *Daphnia galeata* from nine water bodies in the middle and lower reaches of the Yangtze River

Population code	*16S* rDNA							*CO* I gene							Combination of the *16S* rDNA and *CO* I genes						
	*H*	*Hd*	*S*	*π *%	*K*	*Ta*	*Fu*	*H*	*Hd*	*S*	*π *%	*K*	*Ta*	*Fu*	*H*	*Hd*	*S*	*π *%	*K*	*Ta*	*Fu*
HN	2	0.35	5	0.33	1.72	1.06	1.30	5	0.50	13	0.85	3.56	0.37	0.98	6	0.54	18	0.57	5.29	0.65	1.24
AH	3	0.63	6	0.50	2.55	2.05*	1.70*	10	0.77	23	1.57	6.59	0.59	−0.05	12	0.87	29	0.98	9.14	1.02	0.46
TH	4	0.41	7	0.34	1.76	0.11	−0.11	10	0.77	20	0.80	3.37	−1.13	−0.89	14	0.83	27	0.55	5.13	−0.84	−0.74
DS	3	0.33	6	0.28	1.46	−0.07	0.33	7	0.81	9	0.54	2.28	−0.28	0.16	9	0.84	15	0.40	3.75	−0.22	0.26
CH	3	0.21	6	0.17	0.86	−1.12	−0.07	6	0.40	22	0.72	3.03	−1.48	−1.66	7	0.45	28	0.42	3.89	−1.50	−1.37
JS	2	0.13	1	0.02	0.13	−1.16	−1.57	2	0.33	1	0.08	0.33	0.16	0.63	3	0.34	2	0.05	0.45	−0.65	−0.62
LG	2	0.50	1	0.10	0.50	−0.61	−0.48	2	0.50	1	0.12	0.50	−0.61	−0.48	2	0.50	2	0.11	1.00	−0.71	−0.60
PH	3	0.28	2	0.08	0.41	−0.96	−0.66	6	0.60	17	0.61	2.55	−2.16*	−2.94*	6	0.60	19	0.32	2.96	−2.10*	−2.78*
WC	2	0.13	1	0.02	0.13	−1.16	−1.57	3	0.57	2	0.18	0.73	0.55	0.93	4	0.64	3	0.09	0.86	−0.15	−0.08
Overall	7	0.37	9	0.28	1.43	−0.11	0.28	35	0.67	45	0.91	3.81	−1.55	−3.69*	44	0.72	54	0.56	5.247	−1.34	−3.13*

Abbreviations: *Fu*, Fu and Li's *F* value; *Hd*, haplotype diversity; *H*, number of haplotype; *K*, average number of nucleotide differences between whole sequences; *S*, number of segregating sites; *Ta*, Tajima's *D* value; *π*, nucleotide diversity.

* Significance at the 5% level.

In the *16S* rDNA gene sequences of the *D. galeata* populations in nine water bodies in the middle and lower reaches of the Yangtze River, the haplotype diversity ranged from 0.13 to 0.63. The highest haplotype diversity was detected in the AH population (*Hd = *0.63), and the lowest was found in the JS and WC populations (*Hd = *0.13). In the *CO* I gene sequences, a high haplotype diversity (0.33–0.81) was found, and the highest and lowest haplotype diversity appeared in the DS population (*Hd = *0.81) and JS population (*Hd = *0.33), respectively. In the *16S* rDNA gene sequences, the highest nucleotide diversity appeared in AH population (*π* = 0.50%) whereas the lowest occurred in JS and WC populations (*π* = 0.02%). The overall nucleotide diversity *π* of nine *D. galeata* populations was 0.28%. In the *CO* I gene sequences, the highest nucleotide diversity appeared in the AH population (*π* = 1.57%) whereas the lowest occurred in the JS population (*π* = 0.08%). The overall nucleotide diversity *π* of nine *D. galeata* populations was 0.91% (Table 2).

**Figure 3:**
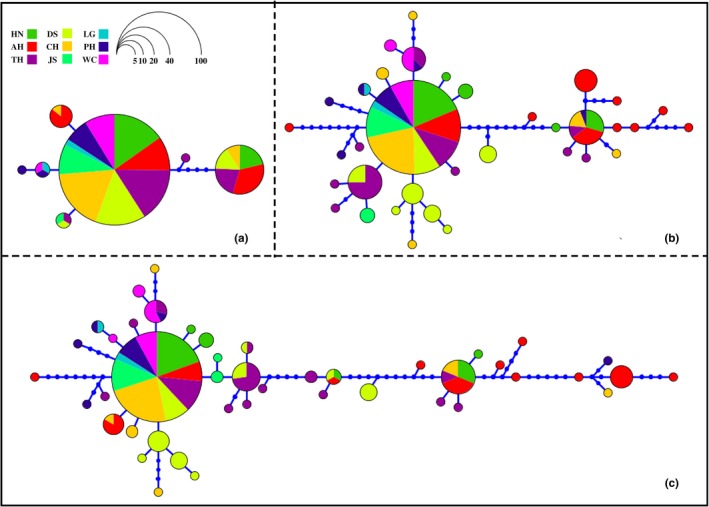
Haplotype networks based on *16S* rDNA, *CO* I gene and their combination of *Daphnia galeata* (a: *16S* rDNA gene sequences, b: *CO* I gene sequences, c: Combined sequences of *16S* rDNA and *CO* I genes. The different colors in the circle represent the populations of different lakes)

Under the combined sequences of the *16S* rDNA and *CO* I genes, 44 haplotypes (Hap1–Hap44) were obtained from the *D. galeata* populations of nine water bodies in the middle and lower reaches of the Yangtze River (Figure 3c), indicating high haplotype diversity (*Hd* = 0.72) and nucleotide diversity (*π* = 0.56%). The highest haplotype and nucleotide diversity all appeared in the AH population (*Hd = *0.87, *π* = 0.98%), whereas the lowest of both diversities occurred in the JS population (*Hd = *0.34, *π* = 0.05%; Table 2). *Fu's Fs* neutral test (*D* = −3.13, *p* < .05) showed that the *D. galeata* populations from the middle and lower reaches of the Yangtze River deviated from neutral evolution. The PH population evidently deviated from the neutral evolution (*Fu's Fs* neutral test: *D* = −2.78, *p* < .05; Tajima's test: *D* = −2.10, *p* < .05). Haplotype networks based on *16S* rDNA, *CO* I gene sequences, and the combination of two gene sequences showed that *D. galeata* individuals from nine water bodies in the middle and lower reaches of the Yangtze River shared the same mitochondrial haplotypes (Figure 3).

Based on the combined sequences of *16S* rDNA and *CO* I genes, among the 44 haplotypes, Hap3 was found in all nine *D. galeata* populations. Hap1 was found in the HN, AH, CH, and TH populations; Hap2, in the HN, AH, and DS populations; and Hap17, in the PH, WC, and TH populations. The remaining haplotypes were only distributed in one to two adjacent populations. Among these haplotypes, the adjacent haplotypes of Hap4–Hap11 and Hap13–Hap15 were distributed in the populations in Huaihe River basin (HN and AH populations, respectively), Hap39 was distributed in the populations in the middle reaches of the Yangtze River (LG and PH populations), and Hap19 and Hap26 were distributed in the populations in the lower reaches of the Yangtze River (TH and DS populations, respectively). Hap12 was mainly located in the AH population although it was also found in the CH population in the junction of the middle and lower reaches of the Yangtze River (Figure 2).

### 3.3 Genetic differentiation of *D. galeata* population in the middle and lower reaches of the Yangtze River

The genetic distances of *D. galeata* populations from nine water bodies in the middle and lower reaches of the Yangtze River ranged from 0.001 to 0.010, and the minimum value appeared in the JS, LG, and WC populations. Meanwhile, the maximum value was obtained in the AH population. Some differences were observed between AH population and other populations, ranging from 0.009 to 0.010 (Table 3).

**Table 3:** Genetic distances between or within populations of *Daphnia galeata* from water bodies in the middle and lower reaches of the Yangtze River based on the combined sequences of *16S* rDNA and *CO* I genes

Population	HN	AH	TH	DS	CH	JS	LG	PH	WC
HN	0.006*								
AH	0.009	0.010*							
TH	0.006	0.010	0.006*						
DS	0.005	0.010	0.005	0.004*					
CH	0.005	0.009	0.005	0.005	0.004*				
JS	0.004	0.009	0.003	0.003	0.003	0.001*			
LG	0.004	0.009	0.004	0.003	0.003	0.001	0.001*		
PH	0.005	0.009	0.005	0.004	0.004	0.002	0.002	0.003*	
WC	0.004	0.009	0.004	0.003	0.003	0.001	0.001	0.002	0.001*

* The average genetic distance within populations.

**Figure 4:**
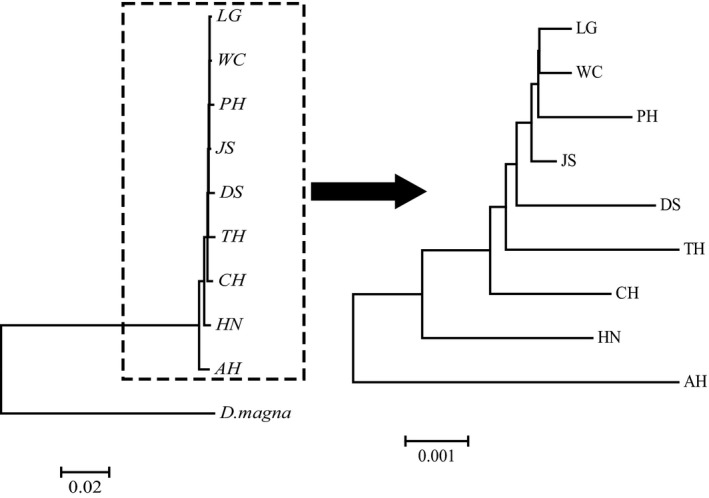
Consensus neighbor‐joining tree of *Daphnia galeata* based on the genetic distance of the combined sequences of *16S* rDNA and *CO* I genes among populations (*Daphnia magna* was used as the outgroup)

**Figure 5:**
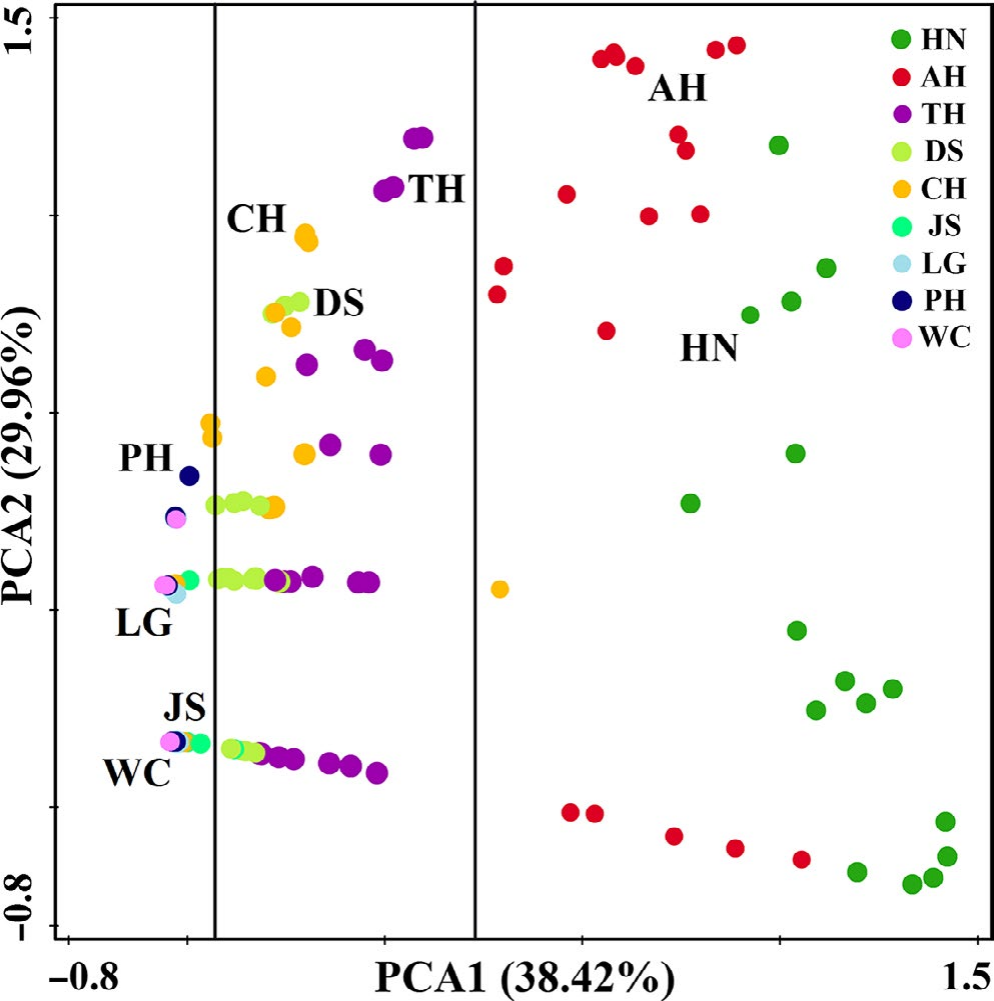
PCA analysis based on the genetic distances of the combined sequences of *16S* rDNA and *CO* I genes within populations

In the neighbor‐joining tree, LG, WC, PH and JS populations of *D. galeata* were clustered into one clade whereas the other five populations were paraphyletic (Figure 4), suggesting that the genetic differentiations of *D. galeata* in the middle and lower reaches of the Yangtze River were common. Additionally, the PCA analysis on the genetic distances of nine *D. galeata* populations displayed a similar pattern (Figure 5). However, those populations with closer distances were respectively gathered into a column. Moreover, in the second axis of PCA, the closer distances appeared in LG, WC, PH and JS populations, suggesting that there were smaller genetic divergences among the individuals within these populations, while the farthest occurred in AH and NH populations, suggesting that there were greater genetic divergences among the individuals within these populations.

AMOVA analysis indicated that the genetic variation of *D. galeata* from nine water bodies in the middle and lower reaches of the Yangtze River mainly existed within populations (85.09%), and significant differences (*p* < .001) was all observed among and within populations (Table 4).

**Table 4:** Molecular variance analysis (AMOVA) of nine *Daphnia galeata* populations from water bodies in the middle and lower reaches of the Yangtze River based on the combined sequences of *16S* rDNA and *CO* I genes

Source of variation	*df*	Sum of squares	Variance components	Variation （%）	*p*	*F*_ST_
Among population	8	93.993	0.39963 Va	14.91	<.001	
Within population	210	478.971	2.28081 Vb	85.09	<.001	
Total	218	572.963	2.68044	100		0.149

Note

Va, Vb are the coefficients of variation.

**Figure 6:**
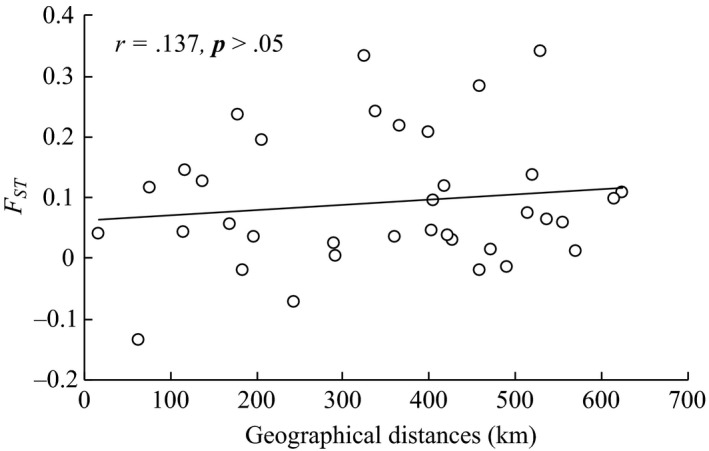
Linear regression of pairwise *F*_ST_ value and geographical distances of nine *Daphnia galeata* populations in the middle and lower reaches of the Yangtze River

In terms of geographical position, Lake Junshan had close distance to Lake Longgan and Wuchang. However, the *F*‐statistics (*F*_ST_) values of the *D. galeata* populations among the JS, LG, and WC populations were all relatively high. Lake Chaohu was far away from Lake Taihu and Dianshan, but the *F*‐statistics (*F*_ST_) values between CH and TH and DS populations were all small. No significant relationship was observed between the *F*_ST_ and geographical distance of nine *D. galeata* populations (*r* = .137, *p* > .05) (Figure 6).

### 3.4 Gene flows of *D. galeata* populations in the middle and lower reaches of the Yangtze River

Based on the genetic differentiation of nine *D. galeata* populations, the effective population size of Group I (LG, WC, PH, and JS populations, from the middle reaches of the Yangtze River) was 502.82, and the effective migration rate (*M*) from Group I to Group ΙΙ (including DS, TH, and CH populations, from the middle and lower reaches of the Yangtze River) in the middle and lower reaches of the Yangtze River was 851.49. The effective migration rate (*M*) was 685.96 from Groups I to ΙΙΙ (including HN and AH populations, from the Huaihe River basin), which are located in the Huaihe River Basin. This finding suggested a strong gene flow from Group I to II and Group ΙΙΙ. The effective population size of Group ΙΙ was 827.31. The effective migration rate (*M*) from Group ΙΙ to Group I was 141.47 and from Group II to Group ΙΙΙ was 402.62. The effective population size of Group ΙΙΙ was 622.56, and the effective migration rates (*M*) from Group ΙΙΙ to Group I was 79.62 and from Group ΙΙΙ to Group ΙΙ was 596.63. Therefore, compared with Group I, the gene flows were relatively weak from Group ΙΙ or Group ΙΙΙ to other groups.

The directional gene flow of nine *D. galeata* populations showed that a strong gene flows from the populations in the middle reaches of the Yangtze River (JS, LG, PH, and WC populations) and the CH population in the junction of the middle and lower reaches of the Yangtze River to the TH and DS populations in the lower reaches of the Yangtze River. In the Huaihe River Basin, a certain degree of genetic communication exists between HN and AH population. The AH and HN populations in the Huaihe River Basin showed strong gene flows with the PH, CH, and TH populations in the middle and lower reaches of the Yangtze River.

**Figure 7:**
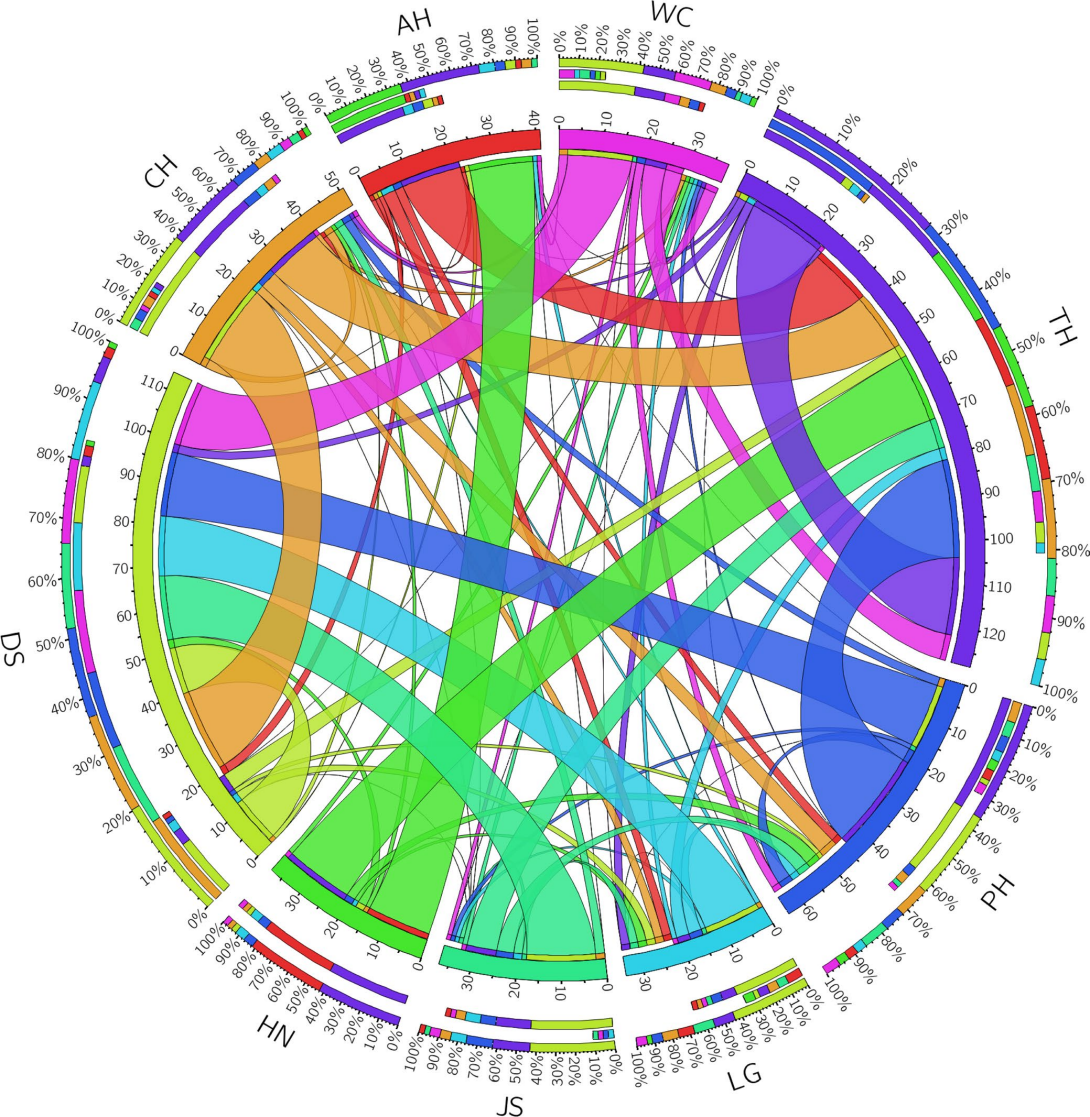
The direction of gene flow of nine *Daphnia galeata* populations in the middle and lower reaches of the Yangtze River based on the combined sequences of *16S* rDNA and *CO* I genes. Different colors represent different populations

In summary, the gene flows of the *D. galeata* populations spread from the middle to the lower reaches of the Yangtze River, and the diffusion of the gene flows from the Huaihe River to the middle and lower reaches of the Yangtze River was also observed (Figure 7).

## 4 DISCUSSION

DNA sequencing has extensively been used as molecular tool on modern taxonomic and biogeographical research (Ratnasingham & Hebert, 2007). Usually, mitochondrial DNA (mtDNA) is relatively conservative, having a strict maternal inheritance. Both *16S* rDNA and *CO* I genes are two popular markers in identifying the genetic differentiation and phylogeny of *Daphnia species* (Ma et al., 2015; Ma, Petrusek, Wolinska, Hu, & Yin, 2019; Wang, Zhang, et al., 2016; Xu et al., 2018, 2014). In the present study, the A + T contents were all higher than the C + G contents in the *16S* rDNA and *CO* I gene sequences of *D. galeata*, which was consistent with other *Daphnia* species (Hebert et al., 2003; Wang, Zhang, et al., 2016; Wu, Wang, et al., 2019a; Xu et al., 2018). Moreover, our results suggested that the combined analysis of the *16S* rDNA and *CO* I gene sequences could clarify more genetic differentiation of *D. galeata* from water bodies located in the middle and lower reaches of the Yangtze River, China. Hebert et al. (2003) found also that the combined analysis based on the *12S* rDNA and *CO* I gene sequences showed a clearer topology on population divergence of *D. ambigua*.

Usually, genetic diversity is referred to the sum of genetic variations of different populations within the same species or different individuals within the same population. The higher the genetic diversity, the stronger the ability of the individuals in the population adapted to environmental changes (Haag et al., 2005). According to Grant and Bowen (1998), the genetic diversity of marine organisms was classified by haplotype diversity (*Hd*) and nucleotide diversity (*π*) of mitochondrial gene sequences. In this study, nine *D. galeata* populations in the middle and lower reaches of the Yangtze River showed high genetic diversity (*Hd* = 0.74, *π* = 0.57%). However, the genetic diversity among populations was significantly different. In Lake Junshan, strong predation pressure of fish evidently inhibited the population density of *D. galeata* (Zhao, Zhang, Peng, Zhang, & Deng, 2018). Owing to its short growing season, the genetic diversity of the *D. galeata* population encountered strong clonal erosion, resulting in low haplotype diversity (Huang, Xu, Xu, & Han, 2017). This phenomenon might be an important reason for low haplotype and nucleotide diversity (*Hd* = 0.34 and *π* = 0.05%) of JS population. Previous reports showed that floods had frequently occurred in the middle and lower reaches of the Yangtze River from 1921 to 2000 (Shi, Jiang, Su, Chen, & Qin, 2004; Xu, Yu, & Ma, 2005). The periodic floods could be accompanied by the rapid expansion and accumulation of mutations of *D. galeata* populations, which affected the genetic diversity of *D. galeata*. *Fu's Fs* neutral test also showed that *D. galeata* populations in the middle and lower reaches of the Yangtze River had historically experienced a bottleneck effect. This finding was consistent with high haplotype diversity and low nucleotide diversity (*Hd* = 0.50–0.84 and *π* = 0.09%–0.48%) of the LG, PH, WC, CH, and DS populations of *D. galeata*. Furthermore, the HN population of *D. galeata* had high haplotype and nucleotide diversity (*Hd* = 0.65 and *π* = 0.70%). Lake Nanhu, which is a small closed lake formed by coal mining collapse, is located in the warm temperate monsoon climate zone. With the continuous deterioration of the lake environment, Lake Nanhu had gradually developed at a eutrophic level (Deng et al., 2010). The polytropic environmental conditions could provide a change for the coexistence or seasonal succession of different *D. galeata* genetic populations and then compensate for the formation of *D. galeata* with high genetic diversity (Xie et al., 2015). Lake Nanhu is also a habitat for some kinds of waterfowls and an important transfer point of some migratory birds in the middle and lower reaches of the Yangtze River (Wang, Jian, Zhang, & Zhou, 2016). Previous studies had indicated that the resting eggs of zooplankton could be attached to the feathers of birds for long‐distance transmission (Figuerola, Green, & Michot, 2005; Proctor, 1964), which played an important role in the gene flow among *Daphnia* populations. Therefore, diverse environmental factors and bird migration might be important reasons for the high genetic diversity of the HN population of *D. galeata*.

Posada and Crandall (2001) believed that ancient haplotypes located at the center of species evolution had the characteristics of high frequency and wide distribution and then spread to other haplotypes. In this study, Hap3 was located not only in the center of haplotype network but also in all nine *D. galeata* populations. Therefore, Hap3 might be regarded as the ancient haplotype in *D. galeata* populations in the middle and lower reaches of the Yangtze River. Notably, the proportion of Hap3 in *D. galeata* populations in the middle reaches of the Yangtze River (LG, WC, PH, and JS) was evidently higher than those found in the lower reaches of the Yangtze River (DS, TH, and CH) and the Huaihe River Basin (HN and AH). This finding might be caused by the spread of *D. galeata* populations. Bohonak and Jenkins (2003) found that species diffusion could promote biological evolution. Zooplankton (e.g., *Daphnia*) and their resting eggs could easily expand to other water bodies through passive diffusions, such as bird migration (Figuerola et al., 2005; Proctor, 1964), fish migration (Mellors, 1975), water diffusion (Michels et al., 2010), and wind transmission (Bilton, Freeland, & Okamura, 2001), and then promote the gene flows among populations. This phenomenon facilitated the spread of *D. galeata* over long distances and rapidly established populations in new habitats. In this study, AMOVA analysis showed that the genetic variation of the *D. galeata* populations mainly occurred within populations, suggesting that gene flows were frequently observed between *D. galeata* populations.

In the present study, the direction of *D. galeata* gene flow is mainly from the middle reaches to the lower reaches of the Yangtze River. This pattern of gene flow was inconsistent with classic pattern which was from an older and more genetically variable population to a younger and less genetically variable population. In several lakes in southern and north‐west Germany, Mantel tests showed a highly significant decrease in Nm (gene flow) with distance for populations of *Daphnia hyalina* (Sabine, 1997). In China, the lower reaches of the Yangtze River had quicker economic development than the middle reaches in past decades. The environmental pollution (especially eutrophication) of the lakes (Lake Chaohu, Lake Taihu, Lake Dingshan) in the lower reaches of the Yangtze River was more serious than those of the lakes (Lake Longgan, Lake Pohu, Lake Wuchang, Lake Junshan) in the middle reaches (Table 1). Higher nutrient concentrations (particularly nitrogen and phosphorous) accelerated the growth and development of phytoplankton, provided more food resources for cladocerans (including *Daphnia*), and then expanded *Daphnia* populations (Deng et al., 2008; Rellstab, Keller, Girardclos, Anselmetti, & Spaaka, 2011). In Swiss lakes, Spaak et al. (2012) thought that environmental changes (i.e., eutrophication) and local adaptation could act together to promote a successful invasion of *D. galeata* in a new habitat. Therefore, increasing nutritional levels could drive the diffuse of *D. galeata* gene flow from the middle reaches of the Yangtze to the lower reaches.

Theoretically, the frequency of individual communication between populations with close geographical distances should be larger than that between populations with far geographical distances. Population segregation or population genetic differences were positively correlated with geographical distances (Hutchinson, 1967). In this study, both neighbor‐joining tree and PCA analysis displayed a similar result, that is, that those populations with closer distances were gathered together. However, except for LG, WC, PH, and JS populations, the other populations were not clustered into a clade. Furthermore, the directional gene flows between populations with close geographical distances were relatively small (such as JS and LG, PH, and WC populations, even AH and CH populations), whereas strong gene flows were observed between populations with farther geographical distances (such as CH and TH, DS populations). The mantel test and linear regression analysis showed that no significant relationship exists between the genetic differentiation and geographical distances among nine *D. galeata* populations in the middle and lower reaches of the Yangtze River (*r* = .137, *p* > .05). The dispersal capabilities of *D. galeata* are high (Louette & De Meester, 2005), in contrast to its high dispersal capacity; however, *D. galeata* has been found to exhibit strong population genetic differentiation, even over small geographical scales (De Meester, 1996). In the middle and lower reaches of the Yangtze River of China, the linkage between the Huaihe River and Yangtze River was changed in 1953 after the construction of Sanhe sluice (Wu, Wang, et al., 2019a), and the interflow of *D. galeata* between the two rivers was also limited. Similarly, Lake Junshan is a typical isolated lake from Lake Poyang, and the connection of Lake Junshan with Lake Poyang and the Yangtze River were cut off after the construction of the lake embankment (Liu, He, et al., 2018). This phenomenon hindered the gene flow of *D. galeata* between populations and led to evident genetic differentiations of *D. galeata* among Lake Junshan and its neighboring lakes. In this study, weak gene exchanges of *D. galeata* between the CH population and the populations in the middle reaches of the Yangtze River were found, whereas strong gene exchanges were observed in the populations in the lower reaches of the Yangtze River. Lake Chaohu is linked to the Yangtze River through the Yuxi River (Wang & Dou, 1998) and could strengthen the gene exchanges between the CH population and the populations in the lower reaches of the Yangtze River. Furthermore, Lake Chaohu and Lake Taihu had the developed navigation function. The water system of Lake Dianshan had also a certain connection with Lake Taihu (Wang & Dou, 1998). Historically, frequent floods had occurred in the middle and lower reaches of the Yangtze River (Shi et al., 2004; Xu et al., 2005). The aforementioned factors in combination can promote gene exchanges and weaken the genetic differentiation of *D. galeata* among the CH, TH, and DS populations. This finding suggests that the geographical distance might not be the main reason for the genetic differentiation of *D. galeata* populations in the middle and lower reaches of the Yangtze River.

Similarly, the genetic differentiation of other *Daphnia* species from water bodies in the middle and lower reaches of the Yangtze River had been observed at different levels (Wang, Zhang, et al., 2016; Wei et al., 2015; Wu, Wang, et al., 2019a). Wei et al. (2015) found that three genetically differentiated *D. galeata* subgroups from eight lakes in the lower reaches of the Yangtze River were observed and did not cluster by their geographical origin in overall. Wang, Zhang, et al. (2016) detected that 10 *D. pluex* populations could initially differentiate into two branches of the middle populations and the lower populations of the Yangtze River. Wu, Wang, et al. (2019a) discovered that seven *D. similoides sinensis* populations were obviously clustered two main clades based on the *CO* I gene sequence, and thought that the habitat fragmentation due to the barrier of the dams and sluices promoted the genetic differentiation and phylogeography of *D. similoides sinensis* in the middle and lower reaches of the Yangtze River. In the present study, except for LG, WC, PH, and JS populations, the other populations were not clustered into a clade. Moreover, there was no significant relationship between *F*_ST_ and geographical distance of nine *D. galeata* populations (*p* > .05). Therefore, the genetic differentiation of *D. galeata* populations in the middle and lower reaches of the Yangtze River might be mainly caused by geographical segregation rather than geographical distance.

## CONFLICT OF INTEREST

None declared.

## AUTHOR CONTRIBUTIONS

Deng Daogui and Liu Qi conceived and designed the experiment. Liu Qi, Zhang Kun, He Ping, Sun Yuchen, Zhang Tingting, Yang Wei, and Liu Wei collected samples. Liu Qi performed the experiment. Liu Qi and Deng Daogui analyzed the sequence data and drafted the manuscript. All authors have read and approved the final manuscript.

## Data Availability

The sequence data of *Daphnia galeata* in this study have been deposited in Dryad Digital Repository (https://doi.org/10.5061/dryad.k0p2ngf41).
